# A dual-mode visual detector for toxic hydrazine†

**DOI:** 10.1039/d1ra03677g

**Published:** 2021-06-28

**Authors:** Brahmjot Kaur, Rameez Raza, Neil R. Branda

**Affiliations:** 4D LABS, Department of Chemistry, Simon Fraser University 8888 University Drive Burnaby BC V5A 1S6 Canada nbranda@sfu.ca

## Abstract

Hydrazine (N_2_H_4_) is one of the commonly used chemical reagents in numerous industries and applications but its toxicity to humans poses a need to develop simple visual detection methods. Herein, we demonstrate a novel dual-mode system to detect and simultaneously consume hydrazine in vapour and solution by using a small photoresponsive molecule that has altered optical response (both colourimetric and fluorescent) after reacting with hydrazine.

## Abstract 2

A small photoresponsive molecule changes colour from blue to colourless when exposed to hydrazine vapours. It also becomes emissive providing two convenient ways of detecting the presence of this toxic chemical.

## Graphical abstract

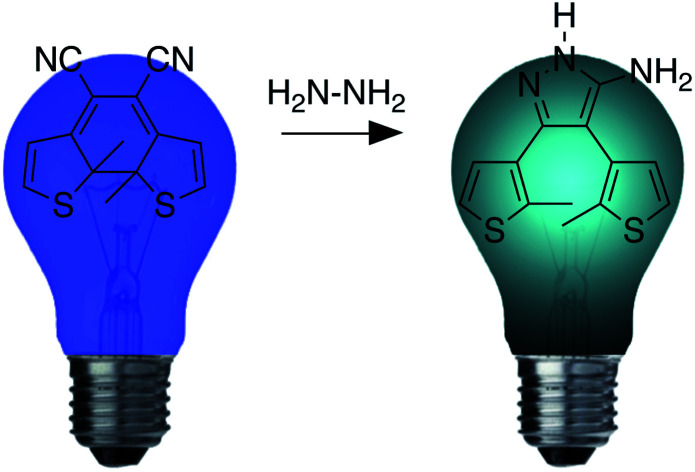

## 1. Introduction

Hydrazine (N_2_H_4_) is classified as a frequently used industrial substrate with applications in sectors including the aerospace industry as rocket fuel propellants,^1,2^ the pharmaceutical industry,^3^ and chemical industries that produce textile dyes and pesticides.^4,5^ Despite hydrazine's growing demand for these and other industries, it is categorized as a likely carcinogen and is toxic for the liver, kidneys and the central nervous system of humans.^5–7^ Humans can be exposed to hydrazine by inhaling its vapours or ingesting water contaminated with it, which can lead to symptoms including irritation of eyes, nose, and throat, dizziness, and nausea.^5^ Hydrazine and its derivatives are also considered as genotoxic impurities that can form methyl adducts with nucleotide bases leading to DNA damage and gene mutations.^8,9^ All of these severe effects have resulted in the US Environmental Protection Agency's (EPA) suggesting a low threshold limit value (∼10 ppb).^7,8^ Even the residual fuel-based debris falling into the oceans after satellite launches has led to severe environmental concerns globally because of hydrazine.^10^ This fact and the steady increase in supply and demand of hydrazine makes it essential to develop simple, user-friendly, and cost-effective methods for detecting this toxic substance.^11,12^

Existing detection methods that employ potentiometry,^13^ ion-selective electrodes,^14^ capillary electrophoresis^15^ metal and metal oxide-based nanostructures^16–18^ rely on the fabrication of tools, technical processes and time-consuming detection. Chromatography-based techniques^4,19–21^ rely on the low volatility and highly polar nature of hydrazine.^4^ These methods also suffer from fundamental problems associated with sophisticated equipment and experiment design, long processing times and greater equipment costs, which limit their use in public places.

Small molecule-based probes can detect hydrazine due to the toxin's nucleophilic behavior by reacting with it to produce a visual change in colour making them potentially more cost-effective and user-friendly.^6,22–36^ While fluorescence-based methods have the advantage of being highly sensitive, an optical response based on a simple change in the colour of a material can be more suitable for ‘naked-eye’ detection. A method that combines both optical techniques would provide a heightened level of reliability.^3,37,38^

Photochromic molecular systems reversibly transform into two or more easily recognizable, differently coloured forms when exposed to different colours of light and have been successfully used as detectors for small analytes.^39^ Those based on the dithienylethene backbone are of specific interest due to their noteworthy optical and thermal properties, and fast response times.^40–44^ These small organic molecules can easily be integrated in bulk materials to act as surface coatings on construction materials and textiles, and would not require any additional electronic controls when making the user aware of the presence of target analytes.

The reported examples of dithienylethene-based photochromic detectors have targeted metal ions,^45–47^ anions,^48,49^ biomolecules^50^ and toxic gases.^51–53^ The example we describe in this report detects hydrazine. Our photoresponsive compound offers a straightforward, easy-to-read visual colour change when exposed to hydrazine. A ‘turn-on’ emission output signal complements the change in colour and provides an additional way to detect this toxic analyte.

**Scheme 1:**
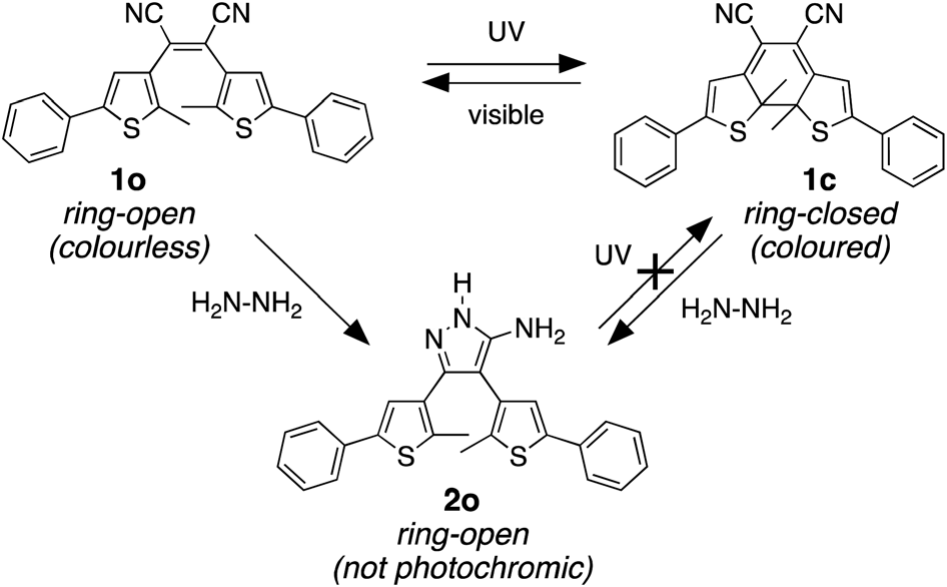
The reversible photoreactions of isomers 1o and 1c. The ring-open isomer (1o) is colourless due to the lack of electronic conjugation between the two heterocycles. The ring-closed counterpart (1c) is coloured because of the extended conjugated π-system running along the backbone. The reaction of either isomer with hydrazine produces 2o, which is not photoresponsive.

Our molecular design was inspired by the reaction of molecular backbones containing nitrile functional groups with hydrazine and the photoresponsive behaviour of the dithienylethene architecture as shown in Scheme 1. Compound 1o contains a photoreactive hexatriene common to all dithienylethenes and undergoes a ring-closing reaction when exposed to UV light to produce isomer 1c. While the ring-open isomer (1o) is colourless, its ring-closed counterpart is coloured due to the extended π-conjugated system running along the backbone of the molecule. Visible light is absorbed by 1c and drives the equilibrium back to 1o resetting the system.

It is well-known that hydrazine readily reacts with substituted cyanoethylenes to produce aminopyrazoles.^54–56^ In our case, both isomers (1o and 1c) react with hydrazine to produce the same aminopyrazole (2o), which is predicted to be non-photoresponsive as already demonstrated for similar pyrazole containing dithienylethenes^57^ and, therefore, is colourless. While the ring-open isomer (1o) is also colourless, it has limited appeal as a visual detector of hydrazine, although as will be shown, it does provide a means of detection using changes in ‘turn-on’ emission. The ring-closed isomer offers a convenient way to detect hydrazine because the blue colour of 1c should disappear when it is converted to 2o. In this manuscript, we describe how both visual changes in colour and emission offer facile methods to detect an important toxin. An additional appeal is that our detector traps hydrazine and removes it from the environment.

## 2. Results and discussion

### 2.1 Synthesis of compounds 1o and 1c

#### 2.1.1 Synthesis of ring-open isomer 1o

**Scheme 2:**
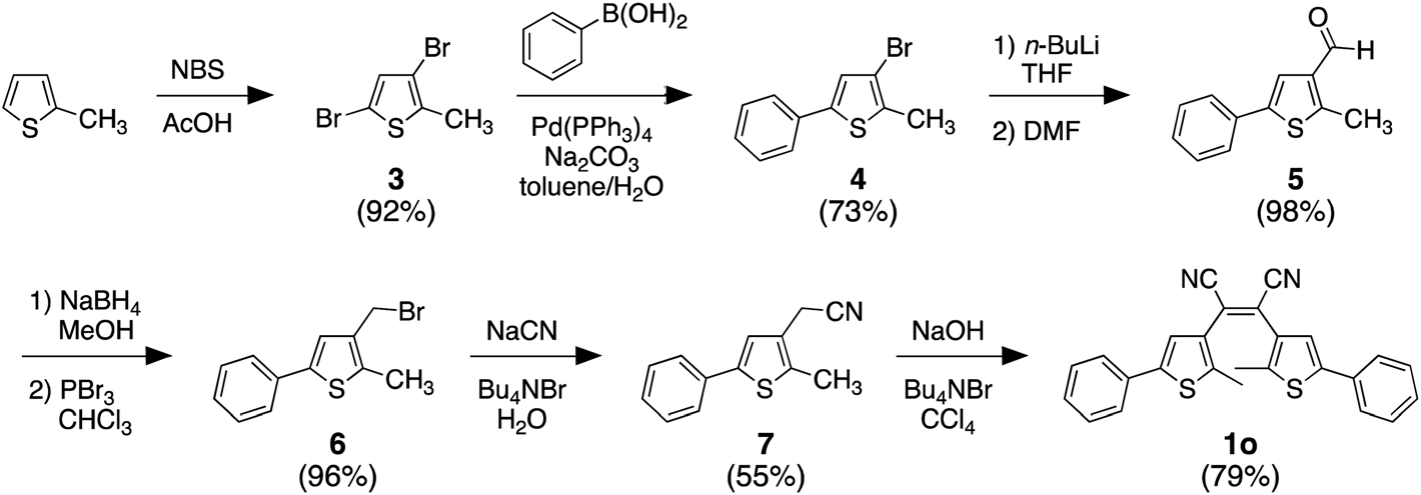
Synthesis of photoresponsive compound 1o.

The photoresponsive dithienylethene (1o) can be synthesized in seven steps from commercially available 2-methylthiophene as described in the literature and as shown in Scheme 2.^58,59^

#### 2.1.2 Photochemistry of ring-open isomer 1o to prepare ring-closed isomer 1c

**Fig. 1:**
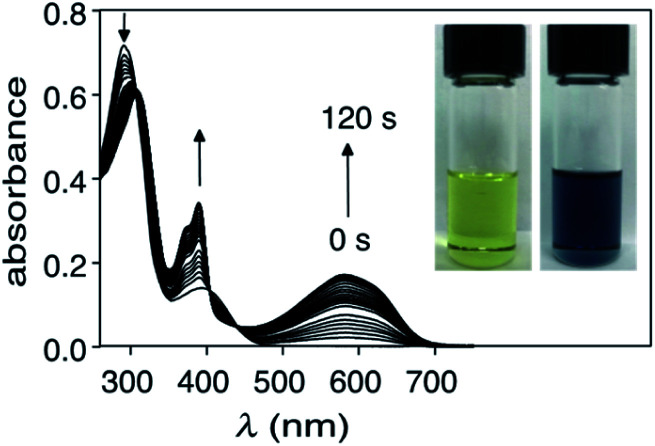
Changes in the UV-vis absorption spectra when a CHCl_3_ solution (1.0 × 10^−5^ M) of ring-open isomer 1o is exposed to 312 nm light for 2 minutes. The inset shows the colour change as a result of the photocyclization.

The photochromism of compound 1o can be visualized by exposing a CHCl_3_ solution of it (1.0 × 10^−5^ M) to a light source centred at 312 nm and measuring the UV-vis absorption spectra during the irradiation. As illustrated in Fig. 1, there is a decrease in the high-energy absorption bands (∼290 nm) and an appearance of a band centred at 580 nm, which accounts for the colour change from pale yellow to deep blue (Fig. 1 inset). At this concentration the system reaches an equilibrium after 2 minutes, at which point the changes in colour stop and the photostationary state is reached. The same photoreaction can also be monitored by ^1^H NMR spectroscopy using CDCl_3_. Naturally, the reaction is significantly slower at the higher concentrations needed for NMR spectroscopy (5 × 10^−3^ M) and is complete after 2 hours. The photostationary state contains 51% of the ring-closed isomer 1c as measured by comparing relative integrals for chemical shifts for each isomer (ESI†). The remaining 49% corresponds to the ring-open isomer 1o. The ring-closed isomer can be isolated by column chromatography using silica gel and 2 : 1 CH_2_Cl_2_/hexanes as the eluant and is stable as long as it is kept away from light.

### 2.2 Treatment of 1c with hydrazine to produce 2o

**Fig. 2:**
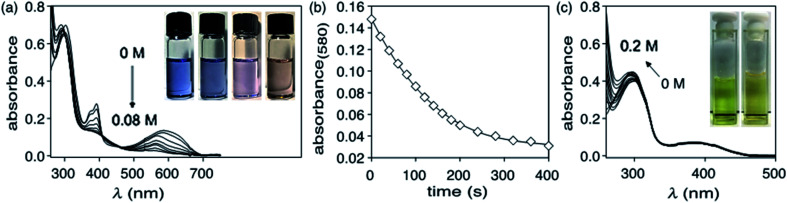
(a) Changes in the UV-vis absorption spectra when a DMSO solution of the isolated ring-closed isomer 1c (4.8 × 10^−5^ M) is treated with increments of 0.5 μL hydrazine monohydrate for a total added volume of 4 μL (equating to a total of 0.08 M N_2_H_4_), (b) the change in intensity of the absorption band at 580 nm when a DMSO solution (4.8 × 10^−5^ M) of the isolated ring-closed isomer 1c is treated with hydrazine monohydrate (0.08 M N_2_H_4_). (c) Changes in the UV-vis absorption spectra when a DMSO solution of the ring-open isomer 1o (9.6 × 10^−6^ M) is treated with increments of 0.5 μL hydrazine monohydrate for a total added volume of 10 μL (equating to a total of 0.20 M N_2_H_4_). The insets in ‘a’ and ‘c’ show the colour change for each experiment.

When a solution of ring-closed isomer 1c in DMSO is treated with hydrazine monohydrate, there is a rapid change in the absorption spectrum and an obvious change in colour of the solution (Fig. 2). In the spectrum, the broad band centred at 580 nm characteristic for the ring-closed isomer disappears and the band at 290 nm increases. From the pseudo-first order plot of these changes, a rate constant of 5.5 × 10^−3^ s^−1^ for the disappearance of 1c can be estimated (see ESI† for details).

The product of the reaction of 1c with hydrazine can be isolated by column chromatography using silica gel and 15% EtOAc in hexanes as the eluant and confirmed as 2o using ^1^H and ^13^C NMR spectroscopy, and FT-IR spectroscopy (ESI†). The most characteristic change when 1c reacts with hydrazine is the disappearance of the band corresponding to C≡N stretching at *ῡ* = 2210 cm^−1^ in the FT-IR spectrum with the simultaneous appearance of a new set of bands corresponding to the amines (N–H stretching at *ῡ* = 3319–3076 cm^−1^, N–H bending at *ῡ* = 1625 cm^−1^, and C–N stretching at *ῡ* = 1160 cm^−1^). In case of ^1^H NMR spectroscopy, the peak corresponding to the thiophene protons of 1c shift downfield from *δ* = 6.72 ppm to *δ* = 7.10 ppm supporting the restoration of aromaticity of the thiophene π-system as a result of ring-opening (for comparison, the thiophene proton in 1o appears at 6.91 ppm in the ^1^H NMR spectrum). The ^13^C NMR results show the disappearance of the peak at *δ* = 115.62 ppm corresponding to the carbon atoms of the nitrile groups and appearance of peaks at *δ* = 117.81 ppm for the bridging carbons of central pyrazole ring and *δ* = 153.24 ppm for carbon atom connected to the NH_2_ group. Tautomer 3o can be ruled out as the product based on density functional theory (DFT) computational methods based, which estimates that it is less stable than 2o (ESI†).
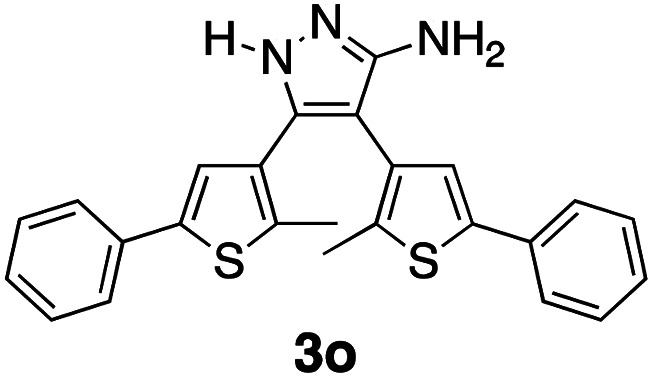


**Scheme 3:**
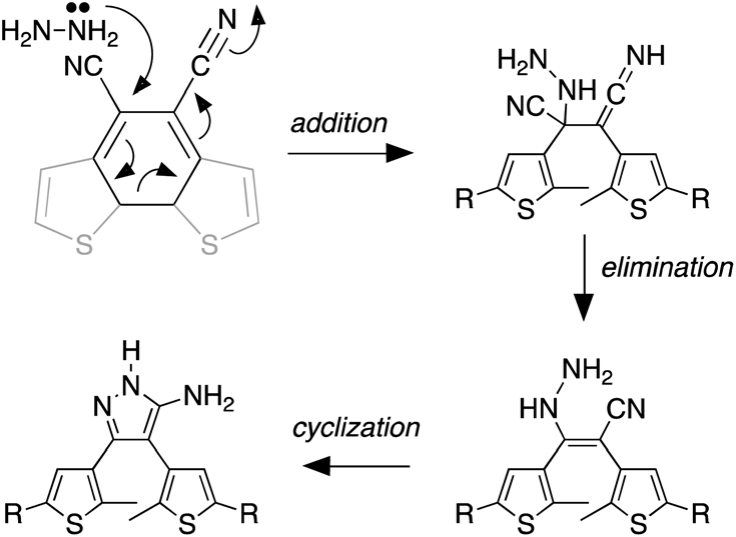
The mechanism that accounts for the ring-opening reaction in the presence of hydrazine. For the full mechanism, see ref. 54–56.

We propose the mechanism shown in Scheme 3 to explain the conversion of the ring-closed isomer (1c) to pyrazole 2o. Initially hydrazine acts as a nucleophile in an addition reaction opening the cyclohexadiene ring system. Elimination followed by cyclization produces the aminopyrazole as described in the literature. The conversion of ring-open 1o to pyrazole 2o follows what has already been reported for cyanoethene derivatives.^54–56^

### 2.3 Limit of detection (LoD) of hydrazine in solution

The limit of detection (the lowest concentration of hydrazine that can be measured with reasonable certainty)^60^ using the ring-closed isomer (1c) can be estimated by treating a solution of 1c (4.8 × 10^−5^ M) in DMSO with a stock solution of hydrazine monohydrate (1.7 × 10^−4^ M) in DMSO in incremental amounts (5 μL) for a total of 110 μL (corresponding to a total concentration of 1.7 × 10^−5^ M N_2_H_4_), and monitoring the decrease in the absorbance at 580 nm (ESI†). The LoD of hydrazine obtained from linear regression is estimated to be 9.0 × 10^−7^ M (29 ppb) (ESI†). The value of limit of detection obtained for probe 1c is much lower than the permissible exposure limit (PEL) for hydrazine determined by the federal Occupational Safety and Health Administration (OSHA) (1 ppm, 8 h time-weighted average),^61^ and the non-disabling and transient acute exposure guideline level (AEGL-1) established by US EPA (0.1 ppm, 8 h time-weighted average).^62^ However, the value obtained remains higher than the TLV recommended by American Conference of Governmental Industrial Hygienists (ACGIH) (0.01 ppm, 8 h time-weighted average).^62^

### 2.4 Changes in emission

**Fig. 3:**
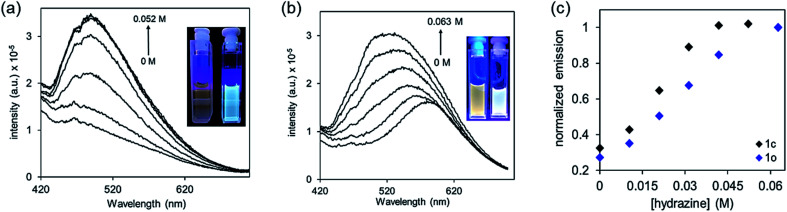
(a) Changes in the emission spectrum when a DMSO solution of the isolated ring-closed isomer 1c (4.8 × 10^−5^ M) is treated with increments of 1.0 μL hydrazine monohydrate for a total added volume of 4 μL (0.052 M N_2_H_4_), (b) a DMSO solution of the ring-open isomer 1o (9.6 × 10^−6^ M) is treated with increments of 1 μL hydrazine monohydrate for a total added volume of 6 μL (0.063 M N_2_H_4_), and (c) the normalized emission for each experiment. The insets show the change in emission for each experiment.

In addition to the conveniently observed change in colour, the photoisomerization process also causes changes in the electronic structure of the molecule that can, in turn, influence the luminescence of 1o and 1c.^63^ Unlike the majority of dithienylethenes, which are weakly emissive in both their ring-open and ring-closed forms, compound 1o is observably emissive due to a localized donor–acceptor intramolecular charge transfer from electron-rich thiophenes to strongly electron-poor nitrile groups (Fig. 3).^64,65^ On the other hand, ring-closed isomer 1c has π-electrons delocalized over the entire molecular backbone and is only weakly emissive.^66,67^ Treating DMSO solutions of either isomer with hydrazine monohydrate results in a substantial increase in emission (Fig. 3), which can also be used as an output signal for the detection.

### 2.5 Detection of hydrazine vapours

**Fig. 4:**
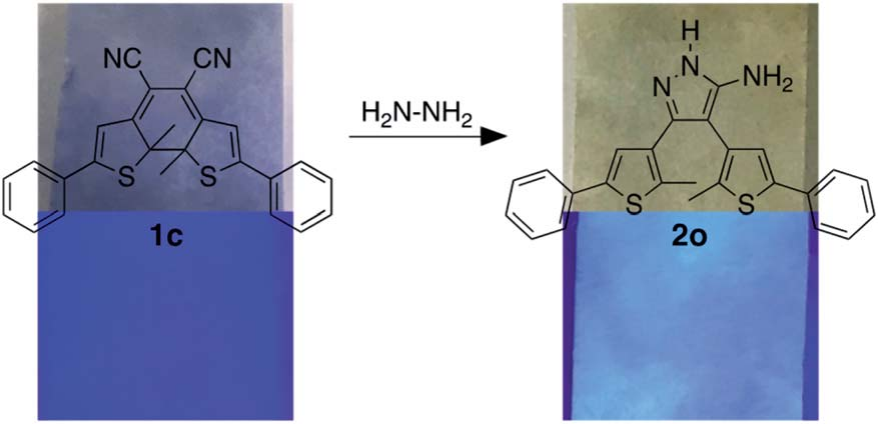
Changes in colour (top) and emission (bottom, *λ*_ex_ = 365 nm) of a piece of filter paper soaked in compound 1c and exposed to vapours of hydrazine.

Visual detection of hydrazine vapour can be readily demonstrated by exposing a piece of filter paper treated with a few drops of a solution of 1c (1 mg) dissolved in 0.1 mL of DMSO, drying the paper in a vacuum oven, placing it elevated in a vial containing 20 drops of hydrazine monohydrate and gently heating to generate hydrazine vapour. The originally blue and non-emissive paper loses its blue colour after a few minutes of exposure and becomes highly emissive under 365 nm light (Fig. 4). When exposed to light of wavelength 365 nm the filter paper remains pale yellow and emissive demonstrating that the non-photoresponsive 2o was generated.

### 2.6 Competitive analytes

The selectivity of compound 1c to hydrazine over other possible interfering analytes such as cations (K^+^, Ca^2+^, Zn^2+^, Na^+^, Mg^2+^, NH_4_^+^), anions (I^−^, Br^−^, CO_3_^2−^, OAc^−^, Cl^−^, SO_4_^2−^), triethylamine (NEt_3_) and phenylhydrazine can be established by carrying out parallel UV-vis absorption experiments. When DMSO solutions of ring-closed isomer 1c (4.8 × 10^−5^ M) are treated with 20 molar equivalents of various analytes in ultrapure water (1.0 × 10^−3^ M) and incubated in dark at 24 °C for 30 min to ensure complete reaction, only the solution treated with hydrazine loses its deep blue colour and has a significant decrease in the intensity of the absorption band centred at 585 nm in the UV-vis absorption spectrum (Fig. 4). Only small changes are observed for the other analytes.

**Fig. 5:**
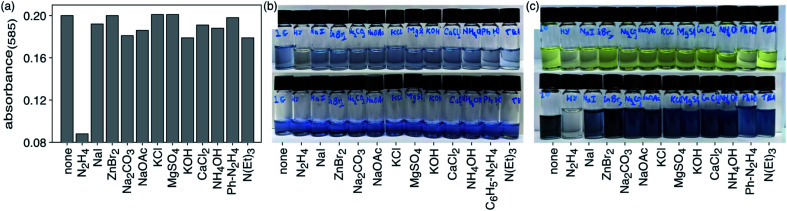
(a) Absorptivities of DMSO solutions of 1c (4.8 × 10^−5^ M) after addition of various analytes. (b) Changes in the colour of DMSO solutions of 1c before (top) and after (bottom) addition of various analytes. (c) Changes in the colour of DMSO solutions of before (top) and after (bottom) addition of various analytes and exposure to 312 nm light for 60 s.

When the identical experiment is carried out with ring-open isomer 1o the solutions containing all the potentially interfering analytes retained their bright yellow colour while the solution containing hydrazine became fainter. When exposed to UV light (312 nm) for 60 s, all the solutions turned blue except for the one containing hydrazine, which demonstrates the selectivity of the detection system (Fig. 5).

## 3. Experimental

### 3.1 Materials and methods

#### 3.1.1 General

All solvents and reagents used for synthesis, chromatography, UV-vis spectroscopy and photochemical studies were purchased from Aldrich, Anachemia, Caledon Labs, Fisher Scientific or Alfa Aesar, and used as received. Solvents used for metal–halogen exchange reactions were dried and degassed by passing them through steel columns containing activated alumina under nitrogen using an MBraun solvent purification system. Hydrazine monohydrate (64–65% hydrazine‡‡WARNING: Hydrazine vapour are toxic upon ingestion or inhalation. Extreme caution must be exercised while handling.) was obtained from Aldrich and used as received. Solvents for NMR analysis were purchased from Cambridge Isotope Laboratories and used as received. Column chromatography was performed using silica gel 60 (230–400 mesh) from Silicycle Inc.

^1^H and ^13^C NMR characterizations were performed on a Bruker Avance-400 instrument with a 5 mm inverse probe operating at 400.13 MHz for ^1^H NMR and 100.61 MHz for ^13^C NMR unless stated otherwise. Chemical shifts (*δ*) are reported in parts per million (ppm) relative to tetramethylsilane using the residual solvent peak as a reference, and splitting patterns are designated as s (singlet), d (doublet), dd (doublet of doubles), t (triplet) and m (multiplet). Coupling constants (*J*) are reported in Hertz. UV-vis absorption spectra were recorded on a Shimadzu UV-3600 Plus spectrophotometer. Fluorescence measurements were performed on a PTI Quantamaster spectrofluorometer. A JDS Uniphase 980 nm laser diode (device type L4-9897510-100M) coupled to a 105 μm (core) fibre was employed as the excitation source. The output of the diode laser was collimated and directed on the 110 samples using a Newport F-91-C1-T Multimode Fiber Coupler. The visible emissions were collected from the samples at π/2 from the incident beam in the plane of the spectrometer. All of the samples were held in a square quartz cuvette (path length of 1 cm). All spectra were corrected for instrument sensitivity. IR spectroscopy measurements were conducted on PerkinElmer Spectrum Two™ IR spectrometer equipped with universal ATR accessory with a 9-bounce diamond top-plate. High Resolution Mass Spectroscopy (HRMS) measurements were performed using an Agilent 6210 TOF LC/MS in ESI-(+) mode. Microanalysis (C, H, N) was carried out using a Carlo Erba EA 1110 CHN Elemental Analyser. Melting points were measured using a Gallenkamp melting point apparatus and are uncorrected.

#### 3.1.2 Photochemistry

All the ring-closing reactions were carried out using the light source from a lamp used for visualizing TLC plates at 312 nm (Spectroline E series, 470 W cm^−2^). The ring-opening reactions were carried out using the light of a 150 W halogen photo-optic source passed through a 435 nm cut-off filter to eliminate higher energy light.

### 3.2 Synthetic methods

Only the final step in Scheme 2 is described below. All other compounds were prepared according to the literature without modifications.^57,58^ The analysis of all compounds matched those in the literature.

#### 3.2.1 Synthesis of 2,3-bis(2-methyl-5-phenylthiophen-3-yl) maleonitrile (1o)

A solution of 2-(2-methyl-5-phenylthiophen-3-yl) acetonitrile (7) (0.93 g, 2.2 mmol) in CCl_4_ (5 mL) was added to a stirring solution of 50% aqueous NaOH (5 mL) containing tetrabutylammonium bromide (35 mg, 0.11 mmol) at room temperature. The mixture was stirred at 50 °C for 1.5 h and the reaction progress was monitored by TLC (10 : 1 hexanes/EtOAc). Upon completion, the reaction mixture was poured into water and extracted with chloroform (2 × 10 mL), dried over MgSO_4_, filtered and concentrated under reduced pressure. Purification by column chromatography using silica gel (10 : 1 hexanes/EtOAc) afforded 735 mg (79%) of the product as a yellow solid.

^1^H NMR (400 MHz, CDCl_3_): *δ* 7.40 (d, *J* = 8.1 Hz, 3H), 7.33 (d, *J* = 7.2 Hz, 2H), 7.29 (d, *J* = 7.1 Hz, 1H), 6.91 (s, 1H), 2.31 (s, 3H).

^13^C NMR (101 MHz, CDCl_3_): *δ* 142.4, 133.1, 131.8, 129.4, 128.6, 127.5, 126.0, 121.1, 116.4, 15.0.

FT-IR (diamond ATR): *ῡ* (cm^−1^) 2924–2851 (C–H stretch, aromatic), 2210 (C≡N stretch), 1445 (C–C stretch, aromatic), 755 (C–H bend, oop), 690 (C–S stretch, thiophene).

Melting point: 132–133 °C.

HRMS (ESI): *m*/*z* (M + H) calculated for C_26_H_18_N_2_S_2_: 423.098417, found: 423.097986.

#### 3.2.2 Photochemical synthesis of ring-closed isomer 1c

A CDCl_3_ solution of compound 1o (5 × 10^−3^ M) in an NMR tube was irradiated with 312 nm light until no further changes were observed in the ^1^H NMR spectrum. After 120 min the photostationary state was reached at this concentration, which contained 51% of the ring-closed isomer 1c as measured by comparing relative integrals for chemical shifts for 1o and 1c. The ring-closed isomer was isolated by column chromatography using silica gel (2 : 1 CH_2_Cl_2_/hexanes).

^1^H NMR (400 MHz, CDCl_3_): *δ* 7.58 (d, *J* = 6.8 Hz, 2H), 7.46 (d, *J* = 6.8 Hz, 2H), 7.44 (d, *J* = 6.7 Hz, 1H), 6.72 (s, 1H), 2.17 (s, 3H).

^13^C NMR (101 MHz, CDCl_3_): *δ* 170.72, 144.69, 130.82, 129.20, 127.25, 115.62, 108.90, 99.58, 57.19, 29.87.

FT-IR (diamond ATR): *ῡ* (cm^−1^) 2924–2851 (C–H stretch, aromatic), 2210 (C≡N stretch), 1445 (C–C stretch, aromatic), 755 (C–H bend, oop), 690 (C–S stretch, thiophene).

#### 3.2.3 Preparation of compound 2o using hydrazine

A solution of 1c (5 mg, 0.012 mmol, 1 equivalent) in DMSO (20 mL) was treated with N_2_H_4_·H_2_O (2 mL, 21 mmol N_2_H_4_, excess). The reaction mixture was stirred at room temperature for 16 h and reaction progress was monitored by TLC (15% ethyl acetate in hexanes). Upon completion, the contents were washed with brine and extracted with ethyl acetate (2 × 20 mL) dried over MgSO_4_, filtered and concentrated under reduced pressure. Purification by column chromatography using silica gel (15% ethyl acetate in hexanes) afforded 4 mg (78% yield) of 2o as a yellow-brown solid.

^1^H NMR (600 MHz, CDCl_3_): *δ* 7.68–7.64 (m, 2H), 7.42 (d, *J* = 9.0 MHz, 3H), 7.10 (s, 1H), 2.11 (s, 3H).

^13^C NMR (151 MHz, CDCl_3_): *δ* 153.24, 133.77, 132.27, 129.14, 129.07, 128.71, 128.62, 127.87, 125.75, 125.60, 117.81, 14.72.

FT-IR (diamond ATR): *ῡ* (cm^−1^) 3319–3076 (N–H stretch), 2924 (C–H stretch, aromatic), 1625 (N–H bend), 1445 (C–C stretch, aromatic), 1160 (C–N stretch), 755 (C–H bend, oop), 690 (C–S stretch, thiophene).

Melting point: 110–113 °C.

Elemental analysis: C = 68.22%, H = 5.32%, N = 8.84%, S = 14.47%.

## Conclusions

In this manuscript, we have described the use of a coloured photoresponsive molecule to conveniently detect toxic levels of hydrazine. The combination of the disappearance of colour and generation of bright emission provides two ways to ensure reliability. Our detector also consumes the hydrazine as it changes its optoelectronic properties, which can be considered as a hydrazine trap if applied to large surface area materials. The advantages of our system are based on a distinct colour change with excellent selectivity (see ESI† for a table of comparative systems).

## Author contributions

B. K. conducted all the experimentation, characterization, prepared the original draft and worked on the revisions. R. R. worked on investigation and validation of the photochemistry experiments and preparation of the first draft. B. K. formulated the idea of the project and developed the methodology. N. R. B. worked on all drafts of the manuscript and supervised the overall project.

## Conflicts of interest

There are no conflicts to declare.

## Supplementary Material

RA-011-D1RA03677G-s001
