# Supplementary material for: A dual-mode visual detector for toxic hydrazine
Source: RSC Adv. 2021 Jun 28;11(37):22835–41. doi: 10.1039/d1ra03677g (PMC9034340; doi:10.1039/d1ra03677g)
Supplement: RA-011-D1RA03677G-s001 [file RA-011-D1RA03677G-s001.pdf]

Electronic Supplementary Material (ESI) for RSC Advances.
This journal is © The Royal Society of Chemistry 2021

Supporting Information

A Dual-Mode Visual Detector for Toxic Hydrazine

Brahmjot Kaur, Rameez Raza and Neil R. Branda*

4D LABS and Department of Chemistry, Simon Fraser University, 8888 University Drive, Burnaby, BC, Canada V5A 1S6

E-mail: nbranda@sfu.ca

^{1}H NMR of ring-open isomer 1o in $CDCl_3$.

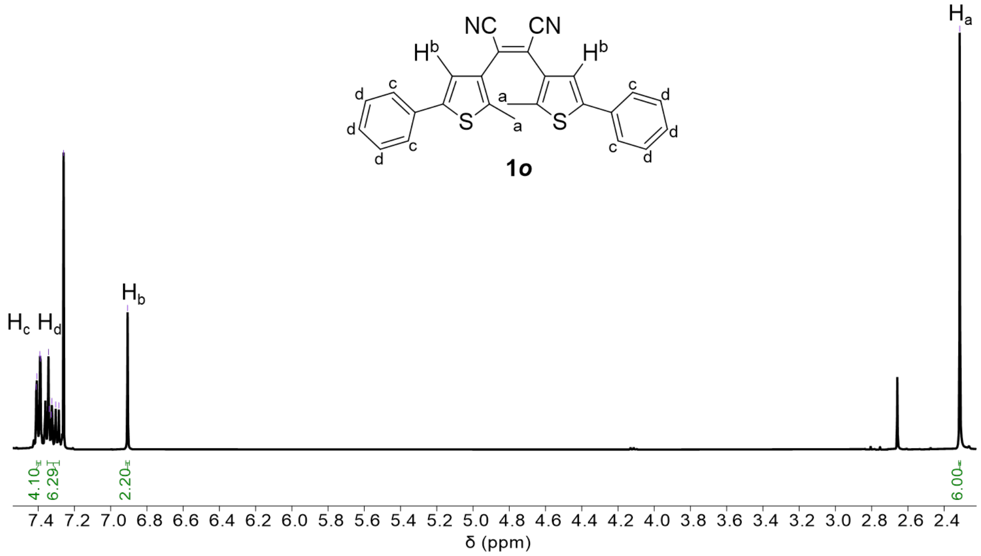

^{1}H NMR of isolated ring-closed isomer 1c $CDCl_3$.

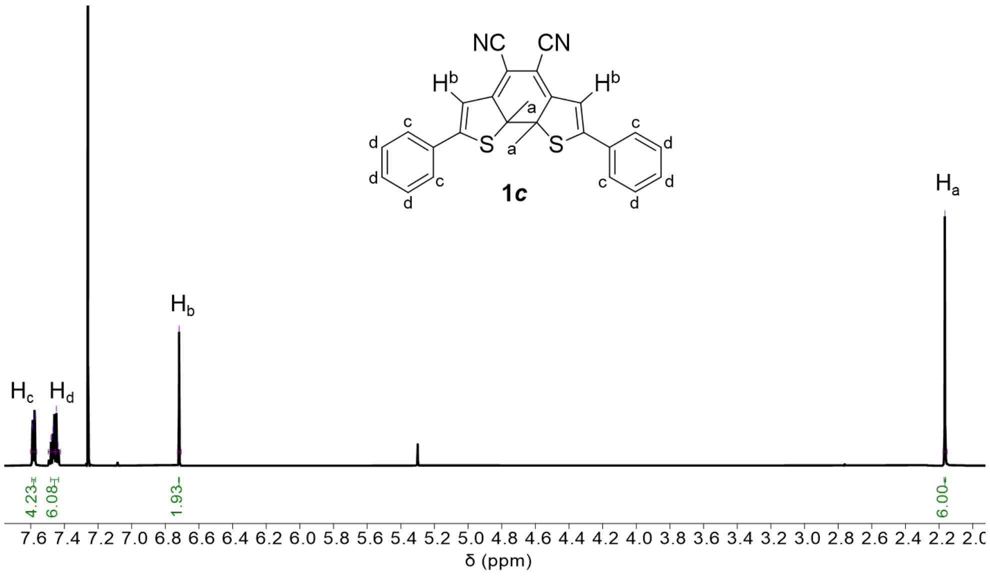

HRMS of isolated ring-open isomer 1o.

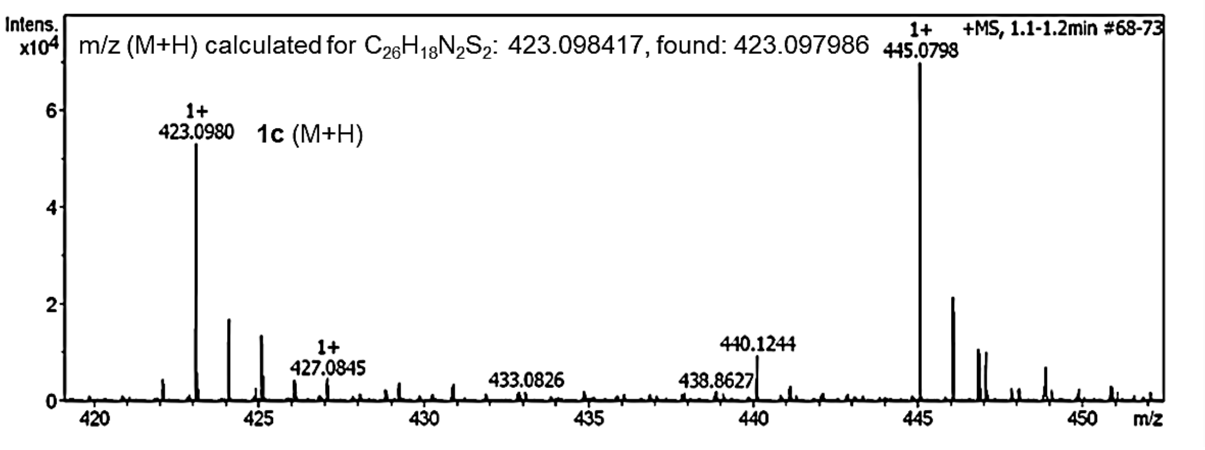

^{1}H NMR of isolated 2o in $CDCl_3$.

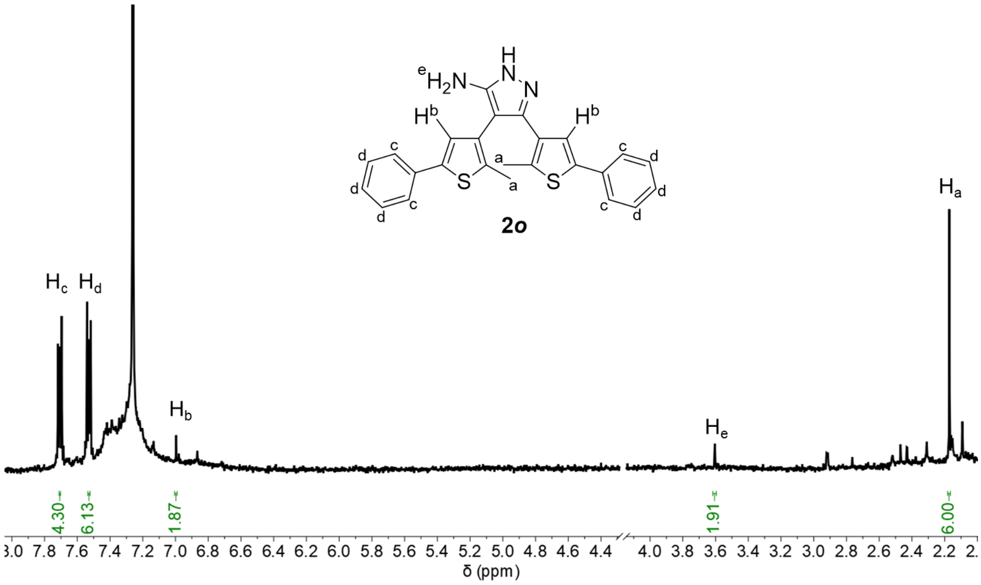

HRMS of isolated 2o.

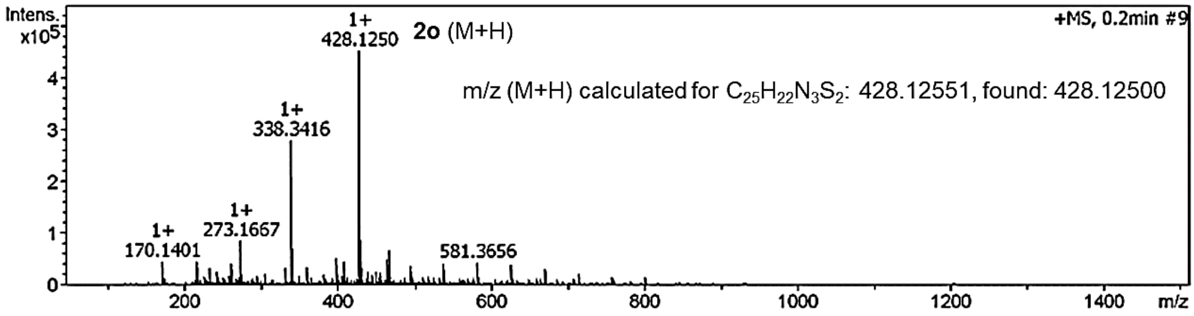

The reaction of hydrazine with ring-closed isomer 1c in solution. A solution of compound **1c** (4.8×10^{-5} M) in DMSO was treated with 4 μL of hydrazine monohydrate (0.08 M) in one portion. The UV-vis absorption spectrum was acquired immediately after the addition, then the cuvette was kept at room temperature in the dark for 400 s, with a spectrum being recorded every 20 s until 200 s, then every 40 s until 400 s. The decrease in the intensity of the band centred at 580 nm corresponding to **1c** was measured over the entire course of the reaction. A pseudo-first order rate constant of 5.5×10^{-3} s^{-1} was estimated from the data. The data are shown in Figure S1.

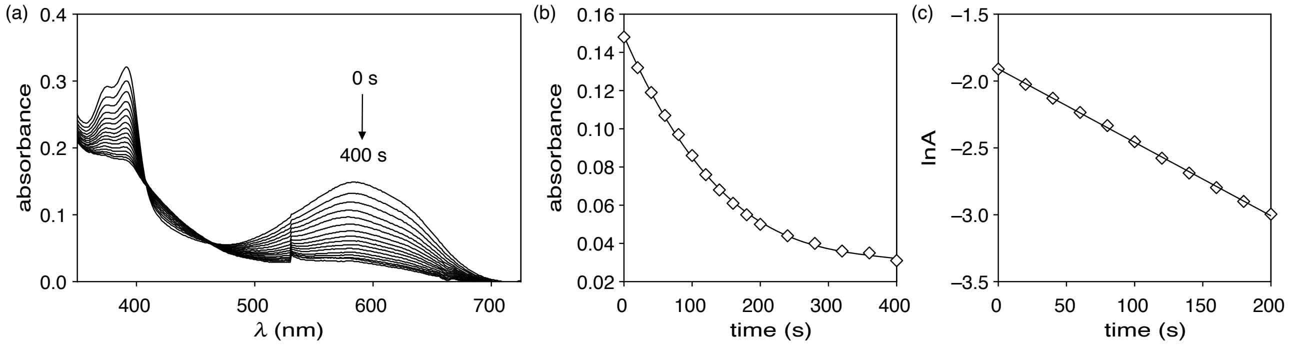

Figure S1. (a) Changes in the UV-vis absorption spectra when a DMSO solution of the isolated ring-closed isomer **1c** (4.8×10^{-5} M) was treated with hydrazine monohydrate (8.0×10^{-2} M). (b) The change in intensity of the absorption band at 580 nm. (c) Pseudo-first order plot of the data presented in 'b'.

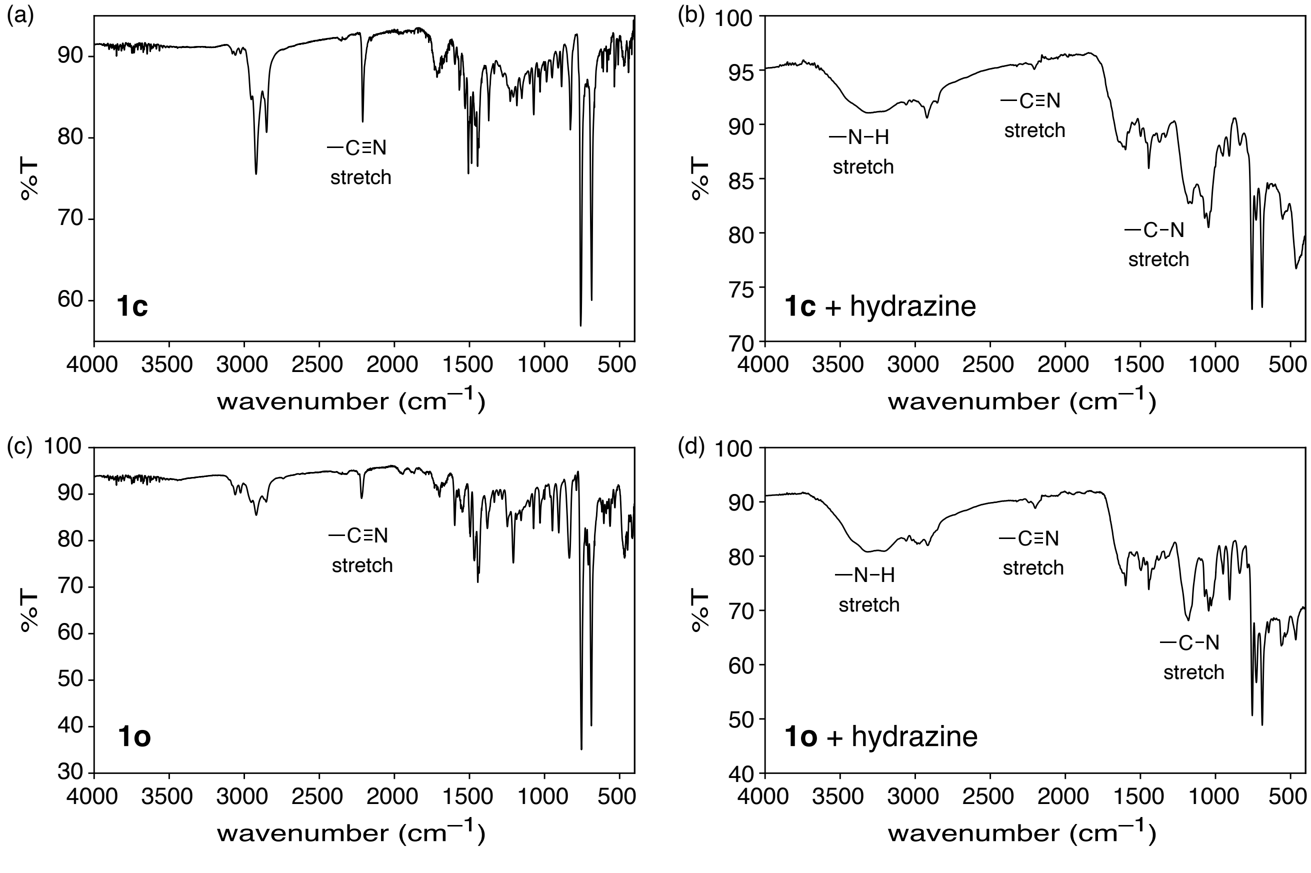

Figure S2. FT-IR spectra of (a) isolated **1c**, (b) the product of **1c** and hydrazine, (c) ring-open isomer **1o**, (d) the product of **1o** and hydrazine.

Calculations of 2o vs 3o. Theoretical calculations were carried out for the total energy of parallel and antiparallel forms of the tautomeric products (**2o** and **3o**) to determine the most likely tautomer and its conformation.

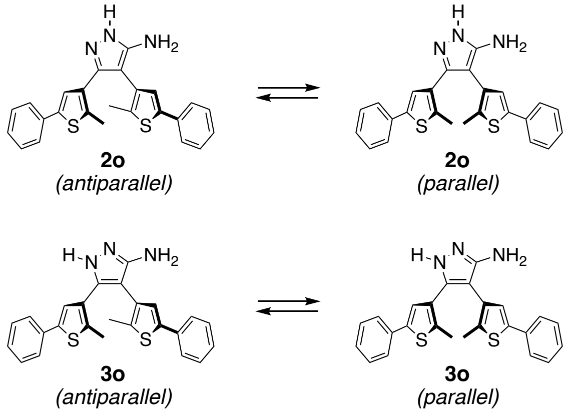

The theoretical value for the relative energy values of all isomers was estimated using computational methods based on density functional theory (DFT) to analyse the electronic or nuclear structure of the molecule in its ground state.[1] The method utilizing B3LYP as the hybrid functional and 6-31G (d) basis set to calculate all the relative energy values was developed in Gaussian 09 software.[2,3,4] Hybrid functional and the basis set used for present calculations have been traditionally reported in the literature for energy calculations in a similar class of molecules.[5] The energy values were obtained in atomic units, a.u. (1 a.u. = 1 Hartree = 2625.4996 kJ/mol).[6] Based on the results of energy calculations, the antiparallel conformers for both **2o** and **3o** were found to have lower energies than their corresponding parallel conformers (Figure S3). The energy difference between the optimized geometries of **2o** (antiparallel) and **3o** (antiparallel) was estimated to be 14.7 kJ/mol. The greater stability of 2*o* can be attributed to the presence of electron-donating -NH_2 group at C-5 position whose lone-pair of electrons are incorporated in the plane perpendicular to the pyrazole ring leading to significant changes in the π-electron distribution in the top 5-amino pyrazole ring.[7,8,9] The lack of photochromic behaviour of the reaction product further verify that structure of the product to be **2o**.[10]

---

[1] P. J. Hasnip, K. Refson, M. I. J. Probert, J. R. Yates, S. J. Clark and C. J. Pickard, *Philos. Trans. R. Soc. A.*, 2014, **372**, DOI:10.1098/rsta.2013.0270.
[2] M. J. Frisch, G. W. Trucks, H. B. Schlegel, G. E. Scuseria, M. A. Robb, J. R. Cheeseman, G. Scalmani, V. Barone, G. A. Petersson, H. Nakatsuji, X. Li, M. Caricato and A. Marenich, Gaussian, Inc., Wallingford CT, 2016.
[3] Density Functional (DFT) Methods, https://gaussian.com/dft/, (accessed 14 May 2020).
[4] Basis Sets, https://gaussian.com/basissets/, (accessed 14 May 2020).
[5] C. T. Poon, W. H. Lam and V. W. W. Yam, *Chem. - A Eur. J.*, 2013, **19**, 3467-3476.
[6] D. L. Lichtenberger, COMMON CONVERSION FACTORS IN COMPUTATIONAL CHEMISTRY, http://u.arizona.edu/~stefanb/linkpages/conversions.html, (accessed 15 May 2020).
[7] A. Secrieru, P. M. O'Neill and M. L. S. Cristiano, *Molecules*, 2020, **25**, 1-28.
[8] I. Alkorta, J. Elguero and J. F. Liebman, *Struct. Chem.*, 2006, **17**, 439-444.
[9] M. Jarończyk, J. C. Dobrowolski and A. P. Mazurek, *J. Mol. Struct. THEOCHEM*, 2004, 673, 17-28.
[10] M. M. Krayushkin, B. V. Lichitskii, A. P. Mikhalev, B. V. Nabatov, A. A. Dudinov and S. N. Ivanov, *Russ J Org Chem*, 2006, **42**, 860-864.

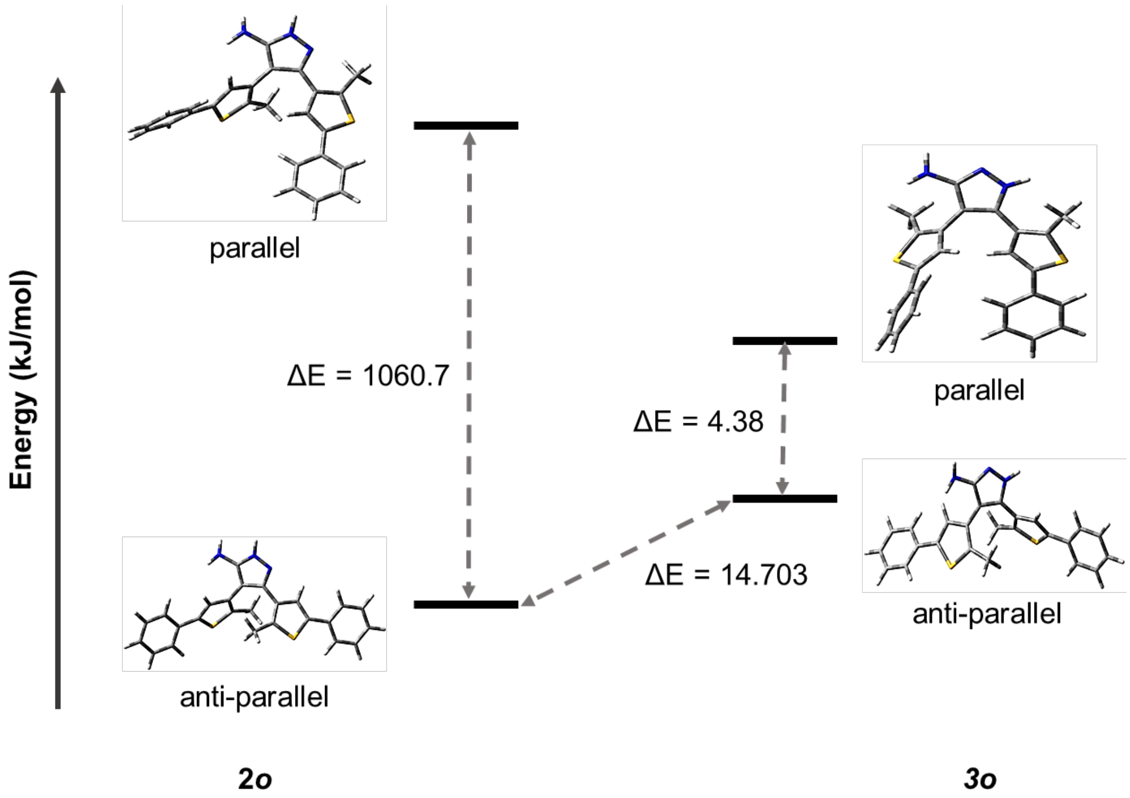

Figure S3. Energy diagram of calculated total energies for all isomers of the product along with the optimized geometries in both parallel and anti-parallel.

Limit of Detection (*LoD*). The limit of detection (the smallest concentration of hydrazine that can be reliably measured[11] using the ring-closed isomer **1c** was estimated by treating a solution of **1c** (4.8×10^{-5} M) in DMSO with stock solution of hydrazine (1.7×10^{-4} M) in DMSO in 5 µL increments for a total of 110 µL (corresponding to a total concentration of 1.7×10^{-5} M N_2H_4), and monitoring the decrease in the absorbance at 580 nm.

The minimum concentration of hydrazine was obtained Using the formula recommended by IUPAC,[12] and other agencies,[13,14,15]

$$LoD = \frac{3\sigma}{m} = \frac{3(2.7 \times 10^{-4})}{8.9 \times 10^{2}} = 9.0 \times 10^{-7}$$

where, σ is the standard error of the y-intercept of the regression line, *m* is the slope of the calibration curve (Figure S4). An *LOD* value of 9.0×10^{-7} M was estimated using this method, which corresponds to 29 ppb of hydrazine.

[11] E. Bernal, in *Advances in Gas Chromatography*, ed. X. Guo, IntechOpen, 2014.
[12] G. L. Long and J. D. Winefordner, *Anal. Chem.*, 1983, **55**, 712-724.
[13] *Guidance for Industry Q2B Validation of Analytical Procedures: Methodology*, 1996, vol. 20857.
[14] J. Ripp, *ANALYTICAL DETECTION LIMIT GUIDANCE & Laboratory Guide for Determining Method Detection Limits*, Madison, 1996.
[15] *Definition and procedure for the determination of the method detection limit—Revision 1.11*, Washington, DC, 2016.

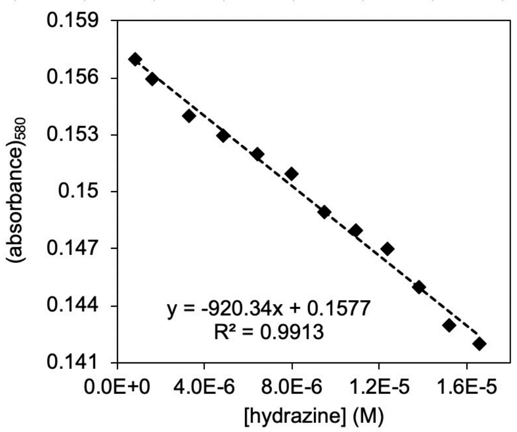

Figure S4. Change in absorbance of at 580 nm when a DMSO solution of **1c** (4.8×10^{-5} M) is treated with hydrazine (8.3×10^{-7} M to 1.7×10^{-5} M).

The reaction of hydrazine with ring-closed isomer 1c in the vapour phase. A piece of filter paper was treated with a few drops of a solution of **1c** (1 mg) dissolved in 0.1 mL of DMSO and dried in a vacuum oven overnight to remove the solvent. The dried filter paper (blue and non-fluorescent) was then placed elevated in a vial containing 20 drops of hydrazine monohydrate ($N_2H_4 \bullet H_2O$) that were placed at the bottom of the vial. The vial was gently heated to generate hydrazine vapour. The blue filter paper became colourless after a few minutes of exposure to the vapour showing that the vapours of hydrazine reacted with **1c** to generate **2o**, which was pale yellow. Images were acquired before and after exposure and are shown in Figure 4 of the manuscript.

The reaction of ring-closed isomer 1c with hydrazine and competing analytes in solution. A solution of **1c** (4.8×10^{-5} M) in DMSO was charged with a total of 1 mL of aqueous solutions (1.0×10^{-3} M) of hydrazine monohydrate, phenyl hydrazine, triethylamine, sodium iodide, zinc bromide, sodium carbonate, sodium acetate, potassium chloride, magnesium sulfate, potassium hydroxide, calcium chloride, ammonium hydroxide and incubated in dark at 24 °C for 30 min. The UV-visible absorption spectrum was acquired after incubation. These results are shown in Fig. 5 of the manuscript.

Comparison with reported small molecule-based probes.

type	reference[a]	solvent	colour change	emissive	*LoD* (M)	vapour sensing
photoswitchable	26	DMSO/water	yellow → pink	yes	1.4×10^{-7}	not reported
	37	EtOH/water	orange → colourless	yes	1.6×10^{-6}	yes
	this work	DMSO	blue → yellow	yes	9.0×10^{-7}	yes
non-photoswitchable	2	DMSO/water	yellow → colourless	yes	0.1×10^{-6}	yes
	23	DMSO/water	colourless → orange	yes	1.3×10^{-7}	yes
	27	DMSO/water	none	yes	1.2×10^{-7}	yes
	28	DMSO	yellow → colourless	yes	81×10^{-9}	not reported
	31	DMSO	purple → yellow	yes	0.7×10^{-9}	not reported

32	acetonitrile/ water	yellow → colourless	yes	1.0×10^{-6}	not reported
33	DMSO/water	purple → yellow	yes	5.8×10^{-8}	not reported
34	EtOH/water	yellow → pink	yes	5.7×10^{-8}	not reported
35	DMF/water	colourless → orange	yes	5.1×10^{-8}	not reported
38	DMSO/water	red → yellow	yes	3.7×10^{-9}	yes

[a] References correspond to those in the manuscript.
